# Vestibular Hearing and Neural Synchronization

**DOI:** 10.5402/2012/246065

**Published:** 2012-03-15

**Authors:** Seyede Faranak Emami, Ahmad Daneshi

**Affiliations:** ^1^Department of Audiology, School of Rehabilitation, Hamadan University of Medical Sciences, Hamadan 16657-696, Iran; ^2^ENT-Head and Neck Research Center, Hazrat Rasoul Akram Hospital, Tehran University of Medical Sciences, Tehran 14455-364, Iran

## Abstract

*Objectives*. Vestibular hearing as an auditory sensitivity of the saccule in the human ear is revealed by cervical vestibular evoked myogenic potentials (cVEMPs). The range of the vestibular hearing lies in the low frequency. Also, the amplitude of an auditory brainstem response component depends on the amount of synchronized neural activity, and the auditory nerve fibers' responses have the best synchronization with the low frequency. Thus, the aim of this study was to investigate correlation between vestibular hearing using cVEMPs and neural synchronization via *slow wave* Auditory Brainstem Responses (*s*ABR). *Study Design*. This case-control survey was consisted of twenty-two dizzy patients, compared to twenty healthy controls. *Methods*. Intervention comprised of *Pure Tone Audiometry* (PTA), *Impedance acoustic metry* (IA), *Videonystagmography* (VNG), *fast wave* ABR (*f*ABR), *s*ABR, and cVEMPs. *Results*. The affected ears of the dizzy patients had the abnormal findings of cVEMPs (insecure vestibular hearing) and the abnormal findings of *s*ABR (decreased neural synchronization). Comparison of the cVEMPs at affected ears versus unaffected ears and the normal persons revealed significant differences (*P* < 0.05). *Conclusion*. Safe vestibular hearing was effective in the improvement of the neural synchronization.

## 1. Introduction

The human vestibule has preserved an ancestral sound sensitivity, which is indicated by the normal findings in the cVEMPs [1–5] and so-called vestibular hearing [6–10]. The vestibular hearing lies in the range of aloud low frequencies (50–800 Hz and above 90 dB spl), which may be obtainable from loud dance music or overt singing [3]. This response can be a physiological basis for the minimum loudness necessary for rock and dance music, so, vestibular hearing contributes to the affective quality of loud sounds [7–10]. Previous study have established that vestibular hearing might be used to assist in the rehabilitation of hearing loss and deafness [3]. Thus, the vestibular hearing can improve the speech processing in the competing noisy conditions, and it can contribute to frequency discrimination of loud tone, and improve speech perception [11]. The range of vestibular hearing happens in the range of the fundamental (*F*_0_) frequency and exceeds that of the cochlea for low frequencies [7]. Indeed, in addition to activating cochlear receptors, low-frequency air conducted sound (ACS) and bone-conducted vibration (BCV) activate vestibular otolithic receptors, ACS only activates saccular afferents, and BCV activates both saccular and utricular afferents [12].

On the other hand, *s*ABR is providing as an estimate of low-frequency (250–500 Hz) sensitivity, Which is comprised of a longer-latency, rounded wave V [13, 14], and is called slow wave negative response (SN10). For some individuals, *s*ABR can achieves amplitude several times of the faster ABR component [14, 15]. It has been suggested that the sharp peak of wave V is generated by the lateral lemniscus as it terminates into the inferior colliculus and that the activity of the inferior colliculus is responsible for the generation of the relatively slow and large negativity following the peak of the wave V. It comes from the side of the brainstem contralateral to the stimulus. The structure of the inferior colliculus must be intact for the generation of wave V. The peak of waveV can be generated by events at this junction [14, 16]. It is known that neurons at the brainstem and primary auditory cortex are responsive to the low frequency [17]. However, the low-frequency components are as important contributors in the neural phenomenons [18] and may serve as the basis for hierarchical synchronization function through which the central nervous system processes and integrates sensory information [19]. Thus, the aim of this research was to investigate correlation between vestibular hearing using cVEMPs and neural synchronization by means of *s*ABR.

## 2. Materials and Methods

### 2.1. Participants

The study involved twenty healthy controls, which consisted of audiology students and hospital staff (10 females and 10 males, mean age 30 years and range 20–39 years). The case group were twenty-two *selected* dizzy patients from subjects who presented with the complaint of disequilibrium (14 females and 8 males, mean age 32 years, and range 20–39 years), which were diagnosed with benign paroxysmal positional vertigo, migraineurs, vestibular neuritis, and psychogenic causes. The dizzy patients were consecutive subjects who presented to the Audiology Department of Tehran University of Medical Sciences (all 42 persons were volunteers). All the subjects received detailed information about the study and the testing that would be involved. Informed consent was obtained from each individual, and the study was approved by Tehran University of Medical Sciences. The exclusion criteria were the history of ear infections and middle ear diseases, which could interfere with cVEMPs measurements.

### 2.2. Recording Procedures

Total of eighty-four ears were evaluated, which had normal otoscopy findings. Testing was performed bilaterally and intervention comprised of *Pure Tone Audiometry *(PTA),* Impedance acoustic metry *(IA), *Videonystagmography *(VNG),* click-evoked or fast wave Auditory Brainstem Response* (*f*ABR), cervical Vestibular Evoked Myogenic Potentials (cVEMPs), and *slow wave* Auditory Brainstem Response (*s*ABR), using the standard devices.

Also, during the process, we ensured that the persons were attended to their task. The social status and sex were not taken into consideration. All of tests performed on same day. In each step of evaluation, when the procedure was completed for the one test, subjects were given a short break and the whole procedure repeated for another.

### 2.3. Pure Tone Audiometry (PTA)

PTA thresholds in the normal range (−10 to 15 dB HL) were obtained from each person's over the frequency range of 250–8000 Hz [20].

### 2.4. Impedance Acoustic Metry (IA)

For the impedance *acoustic*, middle-ear pressure between the limits of ±50 mm H_2_O was evaluated. The values that were out of this limit were omitted from the analyses [21].

### 2.5. Fast Component Auditory Brainstem Response (*f*ABR)

The ABRs to the click stimulation were delivered monaurally with contralateral masking (click = 80 dB SPL: sound pressure level, noise = 50 dB SPL) [13, 15]. We considered the ABR to be abnormal when peaks III and or V were absent or when the peak to peak I–V exceeded the normal limits of our laboratory (4.40 ms for females, 4.58 ms for males). The averaged values that were out of the normal limit were omitted from the analyses.

### 2.6. Videonystagmography (VNG)

VNG was conducted to eliminate the possibility of any additional vestibular pathology. The battery of VNG tests included assessment of the central vestibular and vestibuloocular systems with evaluation of gaze [22].

### 2.7. Cervical Vestibular Evoked Myogenic Potentials (cVEMPs)

During cVEMPs recording patients were instructed to turn and hold their heads as far as possible toward the side contralateral to the stimulated ear [4]. Moreover, one examiner by the finger force on their back head had been keeping the corrected position. The active electrode was placed over the middle portion of the ipsilateral SCM muscle body as this location appears to generate the most reliable and consistent responses. The reference and the ground electrodes were placed over the upper sternum and on the midline forehead, respectively [1]. Auditory stimuli consisted of tone burst (500 Hz, 120 dB peak SPL), rise/fall time = 1 ms, plateau = 2 ms), presented to the ear ipsilateral to the contracted SCM muscle, bandpass filtered (20 Hz to 2 kHz), and a grand average of the 200 responses calculated by a standard evoked potential recorder. The latencies, amplitudes, and peak-to-peak amplitudes of these waves were calculated and recorded [1]. For each subject, the cVEMPs asymmetry ratio (evoked potential ratio) was calculated according to the formula of Murofushi et al.: 100[(*A*_*n*_ − *A*_*d*_)/(*A*_*n*_ + *A*_*d*_)], where *A*_*n*_ = p13 − n23 (the peak-to-peak amplitude in the normal ear) and *A*_*d*_  = p13 − n23 (the peak-to-peak amplitude in the affected ear). In bilateral case cVEMPs asymmetry ratio is not calculated. In the control group, this ratio was calculated using the peak-to-peak amplitudes for the right ear and left ear, respectively. The cVEMPs results for the control group were used as normative data. The normative values for latency and cVEMPs asymmetry ratio were calculated as mean ± two standard deviations [23]. Latencies longer than the calculated upper limit were interpreted as abnormal. Any cVEMPs asymmetry ratio above the calculated upper limit (mean + two standard deviations) was considered to reflect depressed response on the side with lower amplitude findings and was interpreted as abnormal. Absence of a meaningful waveform with p13 and n23 (no response) was also considered an abnormal finding.

### 2.8. Slow Wave ABR Component (sABR)

The subjects were tested without sedation, with noninverting electrode placed at the high forehead and inverting electrode on ipsilateral mastoid and ground electrode on contralateral. Electrode impedances were roughly equivalent and were <5 kilohms at the start of the test. Responses to 2000 stimuli were averaged, and each response (rate of 37/s) was replicated. Responses were filtered from 30 to 3000 Hz. The stimulus in our paradigm was a 2-0-2 tone burst (500-Hz, 120 dB SPL), Blackman windowed. A response window of 25 ms was used when responses were recorded for all toneburst stimuli [14, 15]. The ABR concluded to be abnormal, when peak V was absent or when it exceeded the normal limits of our laboratory.

### 2.9. Analyses

Data were analyzed by *t*-test for equality of means, Levene's test for equality of variances, and one-way ANOVA for continuous variables. A *P*-value of <0.05 was considered to indicate statistical significance.

## 3. Results

### 3.1. Videonystagmography (VNG)

The dizzy patients presented with a total of forty-four ears (%52.2 affected ears or 23 presented with peripheral vestibulopathic and %47.2 unaffected ears or 21 contralateral normal ears). The affected ears consisted of benign paroxysmal positional vertigo (11 ears with BPPV = 25%), migraineurs (5 ears = %11.4), vestibular neuritis (2 ears = %4.4), psychogenic causes with the symptom of true vertigo during few hours after divorce, strife and death of father (5 women-5 ears: %11.4). Twenty-one patients were ipsilesional affected and one patient with BPPV was affected bilaterally.

### 3.2. Cervical Vestibular Evoked Myogenic Potentials (cVEMPs)

Testing of cVEMPs was done in both ears of each control subject (20 right and 20 left ears). The latency and the amplitude values of cVEMPs were detectable in all healthy persons (40 ears safe vestibular hearing). The mean latency values for p13 and n23 were 12.7 ± 1.0 and 20.1 ± 2.2 ms, respectively (Table 1). Therefore the upper limits (mean + two standard deviations) for latency at p13 and n23 in our study were 14.7 and 24.5 ms, respectively. The mean peak-to-peak amplitude in the control group was 25.9 ± 23.8 *μ*v. The mean cVEMPs asymmetry ratio was 6.5 ± 10.2%, and the upper limit for this ratio (two standard deviations above the mean) was 26.9%. 

The cVEMPs abnormalities (insecure vestibular hearing) included both decreased amplitudes and delayed latencies in twelve (1 psychogenic subject, 7 BPPV, 4 migraineurs) and absent responses in eleven (2 vestibular neuritis, 4 psychogenic subjects, 4 BPPV, 1 migraineurs). In all dizzy patients, the cVEMPs asymmetry ratio findings indicated depressed response on the side with lower amplitude findings in a single ear only. The mean p13 and n23 latencies in the affected ears were both longer than the respective means in the control group (Table 1). Also, the differences were significant (*P* < 0.05 for both). The mean peak-to-peak amplitude in the affected ears was significantly lower than that in the control group (*P* < 0.05).

### 3.3. Slow Wave ABR Component (sABR)

*s*ABR was recordable bilaterally from all healthy persons (Table 2). It had lower amplitude (0.94 ± 0.24), rounded shape, and longer latency (6.89 ± 0.42) in the side of lesion (23 affected ears) (*P* < 0.05). Comparison of the sABR at affected ears versus unaffected ears and the normal persons revealed significant differences (*P* < 0.05).

### 3.4. Final Result

The main outcome measures were differences in amplitudes, p13-n23 latencies of the cVEMPs between affected ears (23 ears with insecure vestibular hearing and abnormal *s*ABR) and unaffected ears (21 ears with safe vestibular hearing and normal *s*ABR), respectively.

Comparison of the cVEMPs at affected ears versus unaffected ears and the normal persons revealed significant differences (*P* < 0.05). Thus, safe vestibular hearing improved neural synchronization.

## 4. Discussion

The range of vestibular hearing happens to coincide with the range of our voice *pitch* [8], which varies considerably among men (*F*_0_ = *∼*100 Hz), women (*F*_0_ = *∼*200 Hz), children (*F*_0_ = *∼*400 Hz), [24–26]. *Pitch *is the perceptual correlate of the fundamental frequency (*F*_0_) [16, 26]. The our voice *pitch* or voiced speech sounds and notes from musical instruments often consist of frequencies at integer multiples of *F*_0_. Such sounds, like a single violin note or a syllable in speech, are usually heard during neural synchronization [24, 27–29].

Also, the auditory nerve fibers in the brainstem pathway are temporally precise, with better stimulus synchronization to *F*_0_ [17]. Indeed, the temporal pattern of fibers' responses in the auditory nerve and the cochlear nucleus to medial geniculate body are near periodic, and the frequency of their repetition is synchronized with *F*_0_ [13, 24, 29]. However, the neural synchronization plays a critical role in the transmission of sensory information from the thalamus to the cortex. It is likely that increased synchronization of auditory cortical neurons will similarly enhance the transmission of information to subsequent stages in auditory processing [25, 30, 31]. Thus, the range of vestibular hearing matches with the area of the neural synchronization and the people with safe vestibular hearing have the better interaction in bottom-up processing.

Also, the ABR is composed of several voltage deflections occurring within the first 15 ms after stimulus onset. These deflections (peaks and troughs) represent far-field synchronous activity produced by onset responses of neural elements and abrupt bends in the neural fiber tracts of the eighth nerve and the auditory brainstem pathway. The amplitude of an ABR component depends on the amount of synchronized neural activity and varies with level of stimulation. These factors affect the amount of neural activity generated, the degree of synchronization among neural elements activated, or both. The larger amplitudes of the waves in ABR may represent better auditory fibers that synchronized activation of fewer elements rather than activation of larger numbers of neural elements [13, 15, 32].

Consequently, the major measures of the ABR are the latency and amplitude of its peaks and dependent on synchronization of stimulus onset. The neural synchronization or the neural conduction time in the brainstem pathway is responsible for the peak neural activity. Thus, a change in the transmission time of neural fiber activity results in poorer synchronization and delays in neural activation, leading to longer peak latencies of the combined activity of the neural fibers [13].

Moreover, a number of specific brain areas may be activated by the vestibular hearing [33]. And there is anatomical evidence of a projection from the saccular nerve into the cochlear nucleus. The data available for hearing impaired subjects show some evidence of changes in the pattern of discriminability for tones above vestibular hearing threshold [3].

Finally, we concluded that people with safe vestibular hearing have intact projections to cochlear nucleus, lateral lemniscus, and to inferior colliculus. 

 These projections can increase the peaks of *s*ABR. On the other hand, the lower amplitude, rounded shape, and longer latency of *s*ABR are the evidence for uncertain saccular projections in the brainstem pathway and insecure vestibular hearing. Then, safe vestibular hearing is effective in the improvement of the neural synchronization.

## Figures and Tables

**Table 1:** The mean latency and interpeak amplitude results of Cervical vestibular evoked myogenic potentials in the healthy persons and the dizzy patients.

Subject	p13 Latency (cVEMPs)	n23 Latency (cVEMPs)	Inter-Peak amplitude (*μ*v)
Psychogenic subjects	14.9 ± 1.5	24.8 ± 1.2	25.3 ± 2.1
Benign paroxysmal Positional vertigo	15.12 ± 1.33	24.69 ± 1.19	24.6 ± 1.4
Migraineurs	15.77 ± 1.36	25.33 ± 0.55	23.8 ± 1.9
Vestibular neuritis	Absent	Absent	Absent
Healthy persons	12.7 ± 1.0	22.1 ± 2.2	25.9 ± 23.8

**Table 2:** The mean latency and amplitude slow wave auditory brainstem responses in health Subjects and dizzy patients.

Side	Latency (ms)	Amplitude (*μ*V)
Healthy	5.60 ± 0.47	2.28 ± 0.54
Dizzy	6.89 ± 0.42	0.94 ± 0.24
